# Opioid System Antagonism Alters Vascular Proteome and Collagen Deposition in ApoE^−/−^ Mice

**DOI:** 10.3390/cells14191559

**Published:** 2025-10-08

**Authors:** Kinga Jaskuła, Agata Nawrocka, Piotr Poznański, Aneta Stachowicz, Marzena Łazarczyk, Mariusz Sacharczuk, Dominik S. Skiba

**Affiliations:** 1 Department of Experimental Genomics, Institute of Genetics and Animal Biotechnology, Polish Academy of Sciences, Postępu 36A, 05-552 Jastrzębiec, Poland; k.jaskula@igbzpan.pl (K.J.); a.nawrocka@igbzpan.pl (A.N.); m.lazarczyk@igbzpan.pl (M.Ł.); m.sacharczuk@igbzpan.pl (M.S.); 2 Department of Biotechnology and Nutrigenomics, Institute of Genetics and Animal Biotechnology, Polish Academy of Sciences, Postępu 36A, 05-552 Jastrzębiec, Poland; p.poznanski@igbzpan.pl; 3 Department of Pharmacology, Faculty of Medicine, Jagiellonian University Medical College, 31-008 Krakow, Poland; aneta.stachowicz@uj.edu.pl; 4 Department of Pharmacodynamics, Faculty of Pharmacy, Warsaw Medical University, Banacha 1, 02-697 Warsaw, Poland

**Keywords:** atherosclerosis, opioid system, aorta, naloxone

## Abstract

Atherosclerosis is common cardiovascular disease, leading to complications such as myocardial infarction and stroke. The main causes of these diseases are lipid accumulation and inflammation in large arteries. In this study, we investigated whether opioid receptor blockade impacts factors involved in atherosclerosis development. We administered naloxone to 8-week-old and 36-week-old ApoE^−/−^ mice, then examined the expression of *Col1a1*, and *Col3a1* in the aorta, as well as the influence of naloxone administration on aortic collagen layer thickness and proteomic changes in the aorta. Additionally, we assessed the impact of naloxone on the splenic T-cell populations. The results showed that *Col3a1* expression decreased in young mice but increased in older mice. In 36-week-old mice, naloxone administration led to an increase in aortic collagen layer thickness, but remained unchanged in young mice. Proteomic analysis identified 587 proteins that were altered following naloxone treatment. Our studies suggest that the opioid system is an important factor in atherosclerosis development.

## 1. Introduction

Atherosclerosis is the most common form of cardiovascular disease (CVD), where the main, but not only, components are lipid accumulation and inflammation in large arteries, which can lead to serious clinical complications such as myocardial infarction or stroke [1,2]. Noteworthy, atherosclerosis remains a leading cause of mortality in developed countries [3]. The major players in the development of this disease are endothelial cells (ECs), leukocytes, and intimal smooth muscle cells (SMCs) that are mobilized in response to inflammation and lead to the formation of atherosclerotic plaque [3,4].

In the early stages of atherosclerosis, disturbed blood flow alters ECs, increasing their permeability and allowing plasma low-density lipoprotein (LDL) and triglyceride-rich lipoproteins to enter the vessel wall through trans-endothelial transport or diffusion at cell junctions [5]. Oxidation of these lipoproteins and other inflammatory mediators then activate ECs, resulting in the increased expression of P-selectin, E-selectin, vascular cell adhesion molecule-1 (VCAM-1), and intercellular adhesion molecule-1 (ICAM-1), which further promote the adhesion of leukocytes, especially monocytes, and secretion of chemotactic factors [6,7]. Once entering the intima, monocytes differentiate into macrophages in response to macrophage colony-stimulating factor (M-CSF) and other cytokines [8]. These macrophages are crucial to lesion progression, as shown by the fact that M-CSF-null mice on a hypercholesterolemic background are almost entirely resistant to lesion development [9]. Lesional macrophages internalize modified lipoproteins through scavenger receptors or phagocytose-aggregated lipoproteins, becoming cholesterol-loaded foam cells, which contribute to the enlargement and instability of the atherosclerotic plaque [10,11]. T-cells also play a key role in the pathogenesis of atherosclerosis, contributing to both the initiation and progression of the disease. They are involved in the immune response within the arterial walls, where they recognize modified lipoproteins and activate inflammatory pathways. Specifically, CD4^+^ T-cells can help orchestrate the inflammatory environment, while CD8^+^ T-cells may influence plaque stability. This immune response promotes endothelial dysfunction, smooth muscle cell proliferation, and foam cell formation, all of which contribute to the development of atherosclerotic plaques. Additionally, T-cells can impact the balance between pro-inflammatory and anti-inflammatory responses, influencing disease outcomes and plaque stability [12,13].

In the initial stages of atherosclerosis, collagen is a key component driving vascular remodelling by shaping the extracellular matrix. The vessel wall contains at least 19 types of collagen, while type I and III collagen encoded by *Col1a1* and *Col3a1* genes, respectively, predominate, providing tensile strength and elasticity [14,15]. In the aortic wall, SMCs synthesize collagen and perform a contractile role. During atherosclerotic plaque formation, SMCs migrate from the media to the intima, increasing collagen content [16]. Mesenchymal SMCs migrating to the vascular intima produce extracellular matrix (ECM) proteins such as collagen, and secrete matrix metalloproteinases (MMPs), contributing to ECM deposits arising and further plaque formation [17]. Inflammatory cells, such as macrophages in inflamed atherosclerotic plaques, also release MMPs, causing degradation of matrix collagen and, along with SMCs apoptosis in the intima, impair collagen synthesis. This dynamic imbalance between collagen synthesis and degradation may potentially make the plaque more prone to rupture [18,19].

Atherosclerosis is a chronic inflammatory disease of the arterial wall characterized by lipid accumulation, immune cell infiltration, ECM remodelling, and progressive structural changes in the vasculature. A key component of this process involves interactions between vascular cells and hyaluronan (HA), a major glycosaminoglycan of the ECM that regulates cellular adhesion, migration, proliferation, and inflammatory responses. These HA-mediated processes are especially critical during the early stages of atherosclerosis, when initial immune cell recruitment and subtle structural remodelling begin to shape the nascent lesion environment.

Among the known HA receptors, CD44 has been extensively studied and is recognized for its central role in coordinating immune responses and vascular remodelling during atherogenesis. CD44 mediates leukocyte adhesion and migration into the intima, supports macrophage retention and foam cell formation, and promotes vascular smooth muscle cell (VSMC) proliferation and migration [20]. These actions contribute significantly to early lesion development, even before significant luminal narrowing occurs. Notably, studies using CD44-deficient mouse models have shown reduced lesion formation, decreased inflammatory cell content, and impaired VSMC accumulation, highlighting CD44 as a key regulator of both the immune and structural remodelling components of early atherosclerosis [21].

Building upon this established role of CD44, we turned our attention to another hyaluronan receptor, HMMR (Hyaluronan-Mediated Motility Receptor, also known as RHAMM). While HMMR is known to regulate cell motility, inflammatory signalling, and cytoskeletal dynamics in various biological contexts, its function in atherosclerosis remains largely unexplored [22,23,24]. Given that CD44 and HMMR can mediate both overlapping and distinct pathways in HA signalling, we hypothesized that HMMR may also participate in the initiation and progression of atherosclerotic lesions.

Studies indicate that *Hmmr* expression was absent in uninjured hearts but markedly increased throughout the entire heart following ventricular resection in zebrafish [25]. Similarly, in a rat model of myocardial infarction, neither HMMR nor HA was detected in uninjured hearts; however, both were upregulated and localized in the infarct area within the first few days post-injury [25]. These findings confirm that both *Hmmr* and HA play a role in the formation of scar tissue in response to damage. As we know, atherosclerosis development was believed to be initiated by endothelial injury. It was called the “response to injury” hypothesis [26]. However, nowadays, more factors involved in this process have been determined where the endothelial layer remains undamaged; therefore, the term “endothelial dysfunction” is adopted. Despite this, the atherosclerosis development process has a lot in common with the formation of scar tissue in response to damage.

Another new factor playing a role in vascular remodelling during atherosclerosis may be the opioid system. In Human Aortic Vascular Smooth Muscle Cells (HASMC) β-endorphin treatment (an endogenous opioid peptide) significantly increased smooth muscle cells migration and proliferation [27]. Our data show that naloxone may modify lipid profile in ApoE^−/−^ mice after naloxone treatment and affects proteome profile in the liver [28]. Moreover, our unpublished data indicate a statistically significant increase in *Hmmr* expression in mice with high opioid system activity [29,30] after treatment with the opioid receptor antagonist, naloxone.

The above findings allow us to assume there is a connection between the opioid system and vascular remodelling in early atherosclerosis, as well as a role of *Hmmr* in the progression of this disease. Studying the disease under chow diet conditions allows us to better isolate and understand the specific effects of opioid receptor blockade, without the influence of diet-induced processes [31,32,33].

## 2. Materials and Methods

### 2.1. Animals

All experiments were performed on 8-week and 36-week-old, male B6.129P2-Apoe^tm1Unc^/J (strain. no. 002052) mice on the C57BL/6J background (further referred to as ApoE^−/−^). Animals were housed in groups of 4–5 individuals and maintained in an animal facility of the Institute of Genetics and Animal Biotechnology of the Polish Academy of Sciences, under standard environmental conditions (ambient temperature of 22 ± 2 °C and 55 ± 5% relative humidity) under a 12 h light/dark cycle (lights on at 7 a.m.). Access to tap water and food (LABOFEED H, Kcynia, Poland) was provided *ad libitum*. Study procedures were carried out in accordance with the ethical clearance (permission no. WAW2/093/2024) received from the II Local Ethics Committee for Experiments on Animals in Warsaw.

### 2.2. Drug and Experiment Design

Both 8-week and 36-week-old mice were assigned to either a saline-treated control group or naloxone-treated experimental group. A non-selective opioid system antagonist, naloxone hydrochloride (NLX) (Sigma-Aldrich, St. Louis, MO, USA) was administered. Individuals belonging to the NLX-treated group received daily intraperitoneal injections of freshly prepared NLX dissolved in saline (0.9% NaCl) at a dose of 10 mg/kg for 7 consecutive days. Mice of the control group were administered an equivalent volume of saline.

### 2.3. Measurement of mRNA Expression

RNA from aorta was isolated using TRItidy G (PanReac AppliChem, Darmstadt, Germany) according to manufacturer’s protocol. Total RNA concentration was measured by Nanodrop 2000 (Thermo Fisher Scientific, Waltham, MA, USA). Reverse transcription of RNA was carried out using the High Capacity cDNA Reverse Transcription Kit (Applied Biosystems, Foster City, CA, USA). The expression of *Hmmr*, *Col1a1* and *Col3a1* at mRNA level in the aorta was analyzed using TaqMan^®^ probes (Thermo Fisher Scientific, catalogue numbers: Mm00469183_m1; Mm00802300_m1; Mm00802300_m1, respectively) and the TaqMan^®^ Real-Time PCR Master Mix (Thermo Fisher Scientific). Reactions were performed on 96-well plates using the LightCycler 96 System (Roche Diagnostics, Mannheim, Germany) Real-Time PCR according to standard protocol. Data analysis was conducted using LightCycler 96 Software. Data were normalized to *Tbp* (Mm01277042_m1; Thermo Fisher Scientific) mRNA levels and relative quantification was calculated.

### 2.4. Trichrome Staining

Thoracic aortas were fixed in formalin and dehydrated in increasing series of alcohols (70%, 80%, 96%, 99.8%), two changes each for 30 min at room temperature, followed by clearing in two changes in xylene (Warchem, Warsaw, Poland) for 15 min each. After that, tissues were transferred to mixture of toluene/paraffin (1:1) and incubated for 2 h in 60 °C. In the final step, each aorta was placed in pure molten paraffin overnight and embedded in paraffin blocks. Processed tissue was sectioned on microtome (Hyrax M25, Zeiss, Germany) for 6 µm thick slices and placed on microscopic slides. Before staining, specimens were deparaffinized in two changes in xylene and hydrated in decreasing series of alcohols. Further, trichrome stain was performed with Trichrome Stain (Masson) Kit (Sigma-Aldrich, St. Louis, MO, USA) according to manufacturer’s protocol. Thickness of the collagen layer was determined by averaging 15 measurements taken from one section per subject at equal intervals around the circumference of aorta using ImageJ 1.54g software.

### 2.5. Flow Cytometry

Spleens were harvested and mashed through 70 μm strainers (VWR International, Avantor, Radnor Township, PA, USA) to isolate single cells. RBC lysis buffer (Biolegend, San Diego, CA, USA) was used to deplete red blood cells. Splenocytes were stained in FACS buffer for 20 min at 4 °C in the dark with the monoclonal antibodies (Biolegend, USA). For intracellular staining of CD168 (HMMR) (Proteintech, Rosemont, IL, USA), BD Cytofix/Cytoperm™ Fixation/Permeabilization Solution was used (BD Biosciences, NJ, USA). Cells were analyzed by a CytoFLEX flow cytometer (Beckman Coulter, Brea, CA, USA), and data were analyzed using Flow Jo v.10 (Ashland, OR, USA). For each experiment, fluorescence-minus-one controls (FMO) were performed. In selected experiments, FMO gating strategies were confirmed by isotype controls. To analyze T-cell subpopulations, the gating strategy presented in Figure S1 was applied. Briefly, doublets were excluded by plotting forward scatter height versus area. From the resulting gate, CD3-positive cells were selected to define total T-cells, which were then further subdivided into CD4-positive and CD8-positive subsets. To distinguish naïve, central memory, and effector T-cells in mouse samples, CD44 and CD62L expression was used: naïve T-cells were identified as CD44^−^ CD62L^+^, central memory T-cells as CD44^+^ CD62L^+^, and effector T-cells as CD44^+^ CD62L^−^. CD69-positive cells were gated within the CD4-positive T-cell population. HMMR-positive cells were identified based on histogram plots using FMO (fluorescence-minus-one) controls, and analyzed within CD4^+^, CD8^+^, naïve, central memory, effector, and CD69^+^/CD4^+^ T-cell subsets. A complete list of antibodies used is provided in Supplementary Table S1.

### 2.6. Liquid Chromatography–Tandem Mass Spectrometry (LC–MS/MS) Analysis

Mouse aortas were homogenized using a Tissue Lyser LT (Qiagen, Hilden, Germany) and lysed in a buffer containing 0.1 M Tris-HCl, pH 7.6, 2% sodium dodecyl sulphate, and 50 mM dithiothreitol (Sigma Aldrich, St. Louis, MO, USA) at 96 °C for 10 min. Total protein concentration in lysates and the peptide contents in the digests were assayed using a tryptophan fluorescence-based WF assay [34]. An amount of 70 µg of protein was digested overnight using the filter-aided sample preparation (FASP) method [35] with Trypsin/Lys-C mix (Promega, Madison, WI, USA) (enzyme-to-protein ratio 1:35) as the digestion enzymes. Next, the samples were purified with C18 Ultra-Micro Spin Columns (Harvard Apparatus, Holliston, MA, USA). All samples were dissolved in 0.1% formic acid (FA) at a concentration of 0.5 µg/µL and spiked with the indexed retention time (iRT) peptides (Biognosys, Schlieren, Switzerland). A total of 1 µg of peptide was injected into a nanoEaseTM M/Z Peptide BEH C18 75 µm i.d. × 25 cm column (Waters, Milford, MA, USA) via a nanoEaseTM M/Z Symmetry C18 180 µm i.d. × 2 cm trap column (Waters, Milford, MA, USA) and separated using a 1% to 40% B phase linear gradient (A phase—0.1% FA in water; B phase—80% acetonitrile (ACN) and 0.1% FA) with a flow rate of 250 nL/min on an UltiMate 3000 HPLC system (Thermo Scientific, Waltham, MA, USA) coupled to an Orbitrap Exploris™ 480 Mass Spectrometer (Thermo Scientific, Waltham, MA, USA). The nanoelectrospray ion source parameters were as follows: ion spray voltage: 2.2 kV, ion transfer tube 275 °C. For data-independent (DIA) acquisition, spectra were collected for 145 min. in full scan mode (400–1250 Da), followed by 55 DIA scans using a variable precursor isolation window approach and automatic gain control (AGC) set to custom 1000%. The DIA MS data were analyzed in Spectronaut 19 (Biognosys, Schlieren, Switzerland) [36] software using directDIATM approach. MS data were filtered by 1% false discovery rate (FDR) at the peptide and protein levels, while quantitation was performed at the MS2 level, and global imputation with a missingness rate set to 0.7 was used. Statistical analysis of differential protein abundance was performed at both the MS1 and MS2 levels [37] using unpaired t tests with multiple testing correction after Storey [38,39]. A summary of the quality control for the LC-MS/MS runs is shown in Supplementary Figure S2 (available in link provided after Conclusions paragraph). The mass spectrometry data have been deposited to the ProteomeXchange Consortium via the PRIDE partner repository [40] with the dataset identifier PXD064451.

### 2.7. Statistical Analysis

Firstly, data for all analyses underwent normality check by Shapiro–Wilk test, followed by two-sided Student’s *t*-test to assess changes between saline-treated and NLX-treated groups. Results were considered as statistically significant when *p*-value was less than 0.05. Results presented on graphs are expressed as means ± SD.

## 3. Results

### 3.1. Effect of the NLX Administration on the Expression of Hmmr, Col1a1, Col3a1 in 8-Week-Old Mice

**Figure 1:**
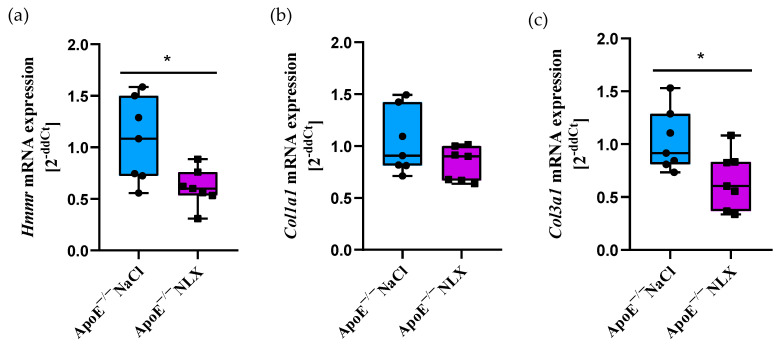
Expression of (**a**) *Hmmr*, (**b**) *Col1a1* and (**c**) *Col3a1* mRNA normalized to *Tpb* in aorta of 8-week-old ApoE^−/−^ mice (*n* = 7 per group) * *p* < 0.05.

NLX treatment in ApoE^−/−^ mice led to a statistically significant decrease in *Hmmr* gene expression (NaCl 1.07 ± 0.41 vs. NLX 0.61 ± 0.18) (*p* = 0.018). We did not observe changes in the expression of the *Col1a1* (NaCl 1.04 ± 0.31 vs. NLX 0.83 ± 0.16); however, administration of NLX caused decreased expression of *Col3a1* (NaCl 1.03 ± 0.29 vs. NLX 0.66 ± 0.27) (*p* = 0.028) (Figure 1).

### 3.2. Effect of the NLX Administration on Collagen Layer Thickness in Thoracic Aorta in 8-Week-Old Mice

**Figure 2:**
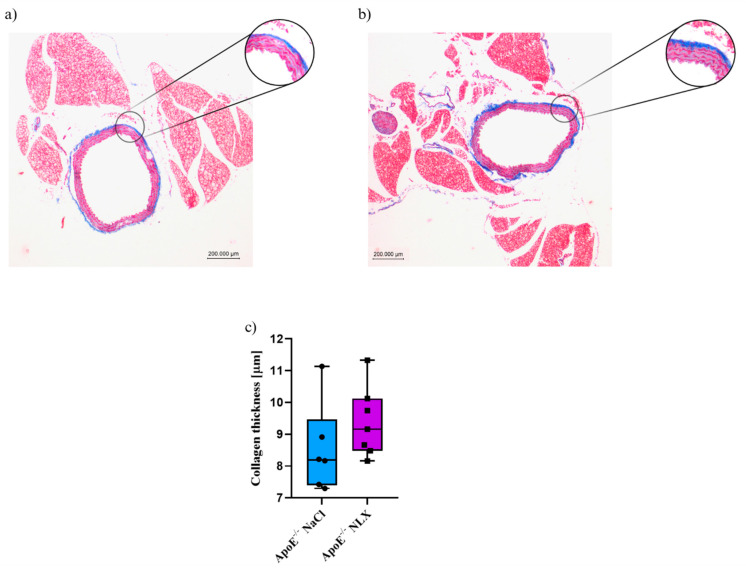
Representative images of Masson’s trichrome-stained thoracic aorta evaluated for collagen thickness in 8-week-old mice after NLX administration. (**a**) Control (*n* = 6); (**b**) treatment group (*n* = 7); (**c**) collagen thickness measured in aorta of control and treated group.

Blockade of opioid receptors with NLX did not cause significant changes in the thickness of the collagen layer in the aorta of 8-week-old mice (NaCl 8.53 ± 1.4 µm vs. NLX 9.38 ± 1.11 µm) (Figure 2).

### 3.3. Effect of the Opioid System Blockade on the T-Cell Subpopulations

**Figure 3:**
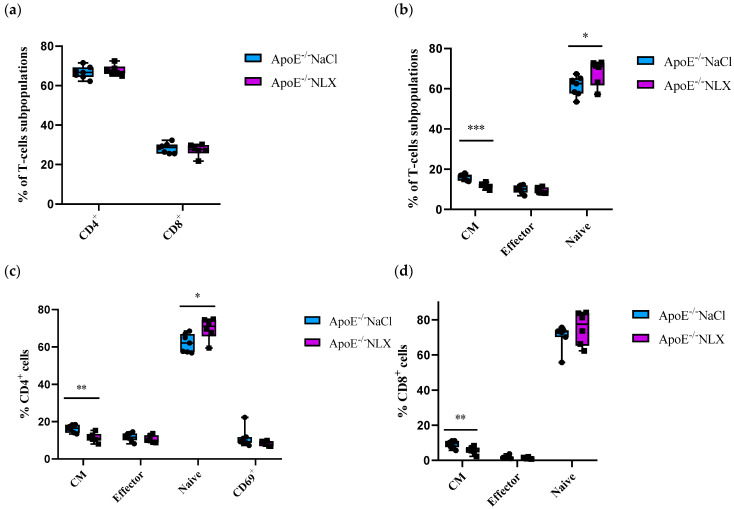
Percentage of T-cell subpopulations (**a**,**b**) isolated from spleen of 8-week-old mice after opioid receptors blockage with NLX. (**c**) Percentage of effector, naive, CM and CD69^+^ cells among CD4^+^ cells. (**d**) Percentage of effector, naive, CM cells among CD8^+^ T-cell subpopulations (*n* = 7 per group), * *p* < 0.05, ** *p* < 0.01; *** *p* < 0.001.

Subpopulations of splenic T-cells collected from 8-week-old ApoE^−/−^ mice after 7 days of the NLX administration were characterized by flow cytometry. Results revealed that the blockade of the opioid system did not affect a percentage of CD4^+^ T-cells (NaCl 66.72 ± 3.09% vs. NLX 67.82 ± 2.59%) and CD8^+^ T-cells (NaCl 28.27 ± 2.55% vs. NLX 27.36 ± 3.05%) (Figure 3a). We observed a statistically significant increase in naive (NaCl 61.25 ± 4.91% vs. NLX 68.06 ± 6.41%) (*p* = 0.031) and central memory (CM) (NaCl 16.19 ± 1.51% vs. NLX 11.72 ± 1.37%) (*p* = 8.22490 × 10^−5^) but not in effector (NaCl 10.03 ± 1.92% vs. NLX 9.21 ± 1.49%) subpopulation (Figure 3b). Administration of NLX did not cause changes in subpopulation of effector (NaCl 11.77 ± 2.23% vs. NLX 10.63 ± 1.96%) and CD69^+^ (NaCl 11.28 ± 5.09% vs. NLX 8.55 ± 1.35%) CD4^+^ T-cells, but we observed statistical increase in naive (NaCl 62.03 ± 4.89% vs. NLX 69.78 ± 5.79%) (*p* = 0.013) and CM (NaCl 16.34 ± 1.96% vs. NLX 11.40 ± 2.45%) (*p* = 0.001) CD4^+^ T-cells (Figure 3c). Moreover, we observed decrease in CM (NaCl 9.04 ± 2.05% vs. NLX 5.75 ± 2.04%) (*p* = 0.007) CD8^+^ T-cells but not in naive (NaCl 70.75 ± 6.83% vs. NLX 75.25 ± 9.39%) or effector (NaCl 1.78 ± 0.87% vs. NLX 1.28 ± 0.49%) CD8^+^ T-cells (Figure 3d).

**Figure 4:**
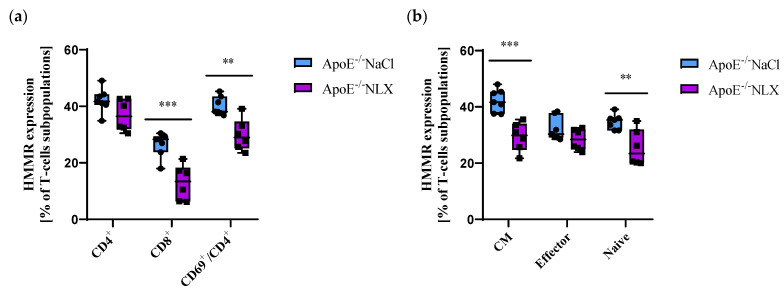
(**a**) *Hmmr* expression on CD4^+^, CD8^+^ and activated CD4^+^ T-cells. (**b**) *Hmmr* expression on effector, naive, and CM subpopulation of T-cells (*n* = 7 per group); ** *p* < 0.01; *** *p* < 0.001.

NLX treatment did not affect *HMMR* expression on CD4^+^ T-cells (NaCl 42.15 ± 4.29% vs. NLX 36.88 ± 5.57%) but decreased its expression on CD8^+^ T-cells (NaCl 26.46 ± 4.30% vs. NLX 13.00 ± 6.25%) (*p* ≈ 0.0008) and CD69^+^/CD4^+^ T-cells (NaCl 40.10 ± 3.31% vs. NLX 29.90 ± 5.64%) (*p* = 0.001) (Figure 4a). We also observed decreased *HMMR* expression in CM (NaCl 42.26 ± 4.03% vs. NLX 29.37 ± 5.09%) (*p* = 0.0003) and naive (NaCl 34.75 ± 2.66% vs. NLX 25.50 ± 6.36%) (*p* = 0.007) but not in effector (NaCl 32.18 ± 4.19% vs. NLX 28.43 ± 3.75%) subpopulations (Figure 4b).

### 3.4. Effect of the NLX Treatment on the Expression of HMMR Hmmr, Col1a1, Col3a1 in 36-Week-Old Mice

**Figure 5:**
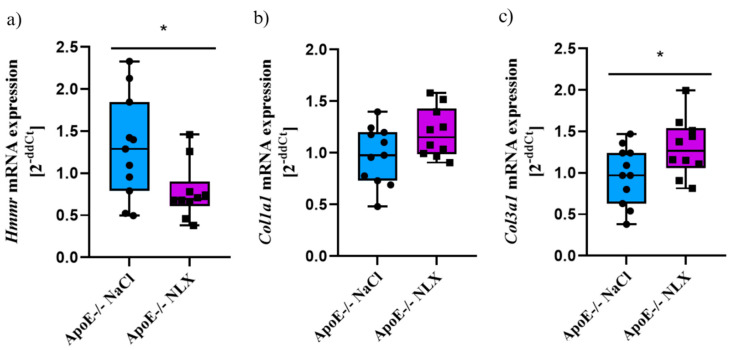
Expression of (**a**) *Hmmr*, (**b**) *Col1a1* and (**c**) *Col3a1* mRNA normalized to *Tbp* in aorta of 36-week-old ApoE^−/−^ mice (NaCl *n* = 11; NLX *n* = 10) * *p* < 0.05.

After NLX administration, we observed a statistically significant decrease in *Hmmr* gene expression in comparison to control group (NaCl 1.29 ± 0.61 vs. NLX 0.78 ± 0.33) (*p* = 0.028). We did not observe changes in the expression of the *Col1a1* gene (NaCl 0.98 ± 0.28 vs. NLX 1.19 ± 0.24); however, NLX treatment led to significant decrease in *Col3a1* gene expression (NaCl 0.97 ± 0.35 vs. NLX 1.31 ± 0.35) (*p* = 0.042) (Figure 5).

### 3.5. Effect of the NLX Administration on Collagen Layer Thickness in Thoracic Aorta in 36-Week-Old Mice

**Figure 6:**
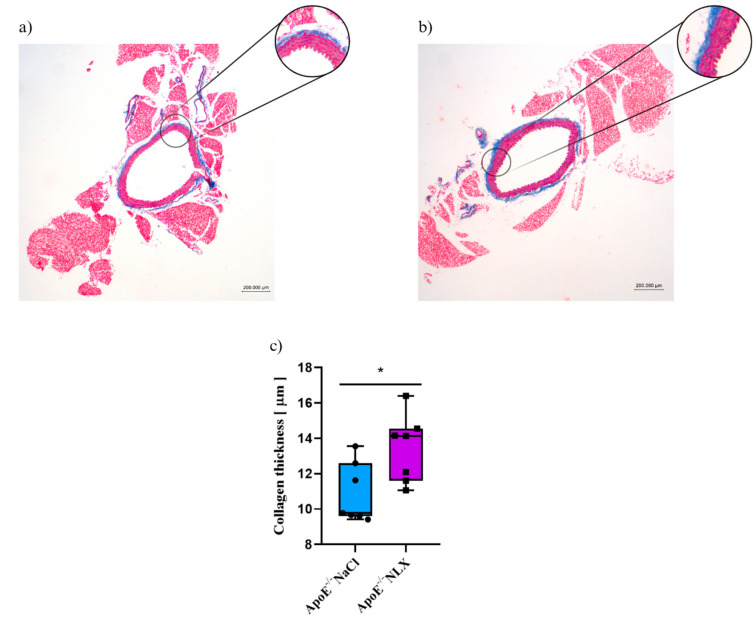
Representative images of Masson’s trichrome-stained thoracic aorta evaluated for collagen thickness in 36-week-old mice after NLX administration. (**a**) control (**b**) after NLX treatment (**c**) collagen thickness measured in aorta of control and NLX-treated group (*n* = 8 per group), * *p* < 0.05.

Blockade of opioid receptors with NLX resulted in alterations in the thickness of the collagen layer in the aorta as shown by a significant *t*-test result (*p* = 0.022). Measurement of the collagen layer revealed that it was significantly thicker in mice treated with NLX (13.43 ± 1.91 µm) compared to the NaCl (control) group (10.89 ± 1.69 µm)(Figure 6).

### 3.6. Effect of the Opioid System Blockade on the T-Cell Subpopulations

**Figure 7:**
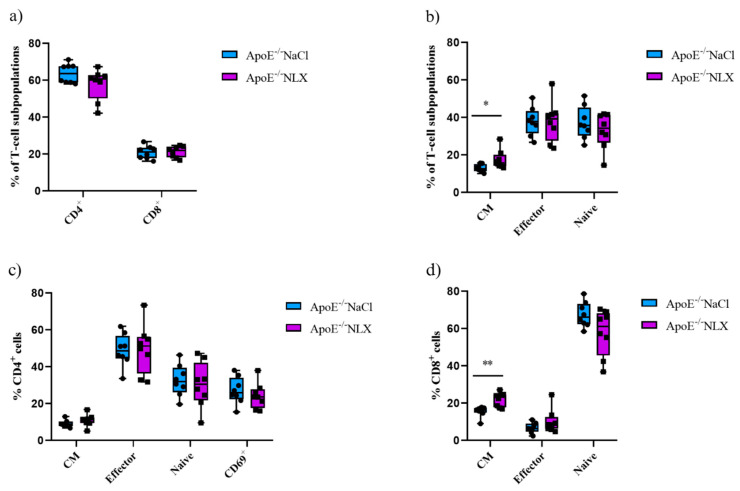
Percentage of T-cell subpopulations (**a**,**b**) isolated from spleen of 36-week-old mice after blockage of the opioid receptors. Percentage of effector, naive, CM, and CD69^+^ cells among CD4^+^ cells. (**c**) Percentage of effector, naive, CM cells among CD8^+^ cells. (**d**) T-cell subpopulations (*n* = 8 per group) * *p* < 0.05; ** *p* <0.01.

Subpopulations of splenic T-cells from 36-week-old ApoE^−/−^ mice after 7 days of the NLX administration were characterized by flow cytometry. Results revealed that the blockade of the opioid system did not affect a percentage of CD4^+^ T-cells (NaCl 63.73 ± 5.25% vs. NLX 57.82 ± 8.54%) and CD8^+^ T-cells (NaCl 20.93 ± 3.57% vs. NLX 21.28 ± 3.05%) (Figure 7a). We also did not observe changes in naive (NaCl 37.24 ± 8.72% vs. NLX 32.95 ± 9.52%) or effector (NaCl 37.97 ± 7.56% vs. NLX 37.98 ± 10.85%) subpopulations but we observed statistically significant increase in CM subpopulation (NaCl 12.93 ± 2.00% vs. NLX 17.61 ± 5.04%) (*p* = 0.028) (Figure 7a). Administration of NLX did not cause changes in subpopulation of effector (NaCl 49.06 ± 8.82% vs. NLX 49.86 ± 13.42%), naive (NaCl 32.63 ± 8.52% vs. NLX 30.20 ± 12.43%), CM (NaCl 9.20 ± 1.91% vs. NLX 10.97 ± 3.25%), and CD69^+^ (NaCl 27.10 ± 7.33% vs. NLX 24.18 ± 7.02%) CD4^+^ T-cells (Figure 7b). However, we observed increase in CM (NaCl 15.60 ± 2.85% vs. NLX 21.96 ± 3.81%) (*p* = 0.002) CD8^+^ T-cells but not in naive (NaCl 67.47 ± 6.73% vs. NLX 57.81 ± 12.50%) or effector (NaCl 6.71 ± 2.73% vs. NLX 10.22 ± 6.36%) CD8^+^ T-cells (Figure 7c).

**Figure 8:**
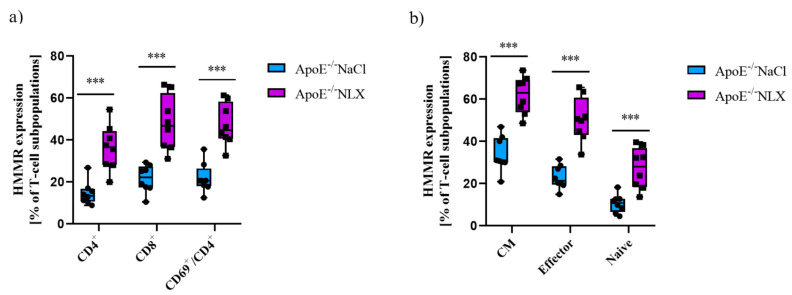
(**a**) HMMR expression on CD4^+^, CD8^+^, and activated CD4^+^ T-cells. (**b**) HMMR expression on effector, naive, and CM subpopulation of T-cells (*n* = 9 per group) *** *p* < 0.001.

Furthermore, NLX administration affected HMMR expression on CD4^+^ T-cells (NaCl 14.63 ± 5.61% vs. NLX 36.30 ± 10.91%) (*p* ≈ 0.0002), CD8^+^ T-cells (NaCl 21.58 ± 6.49% vs. NLX 47.97 ± 13.15%) (*p* ≈ 0.0002), and CD69^+^/CD4^+^ T-cells (NaCl 21.84 ± 7.09% vs. NLX 47.34 ± 10.10%) (*p* = 4.24152 × 10^−5^) (Figure 8b). We also observed statistically significant increase in HMMR expression in CM (NaCl 34.00 ± 8.36% vs. NLX 61.89 ± 8.95%) (*p* = 1.54018 × 10^−5^), naive (NaCl 10.36 ± 4.35% vs. NLX 27.24 ± 9.77%) (*p* ≈ 0.0006), and effector (NaCl 23.06 ± 5.54% vs. NLX 50.31 ± 10.56%) (*p* = 1.49284 × 10^−5^) subpopulations (Figure 8a).

### 3.7. Impact of NLX Administration on the Proteomic Profile of Aortas in 36-Week-Old Mice

**Figure 9:**
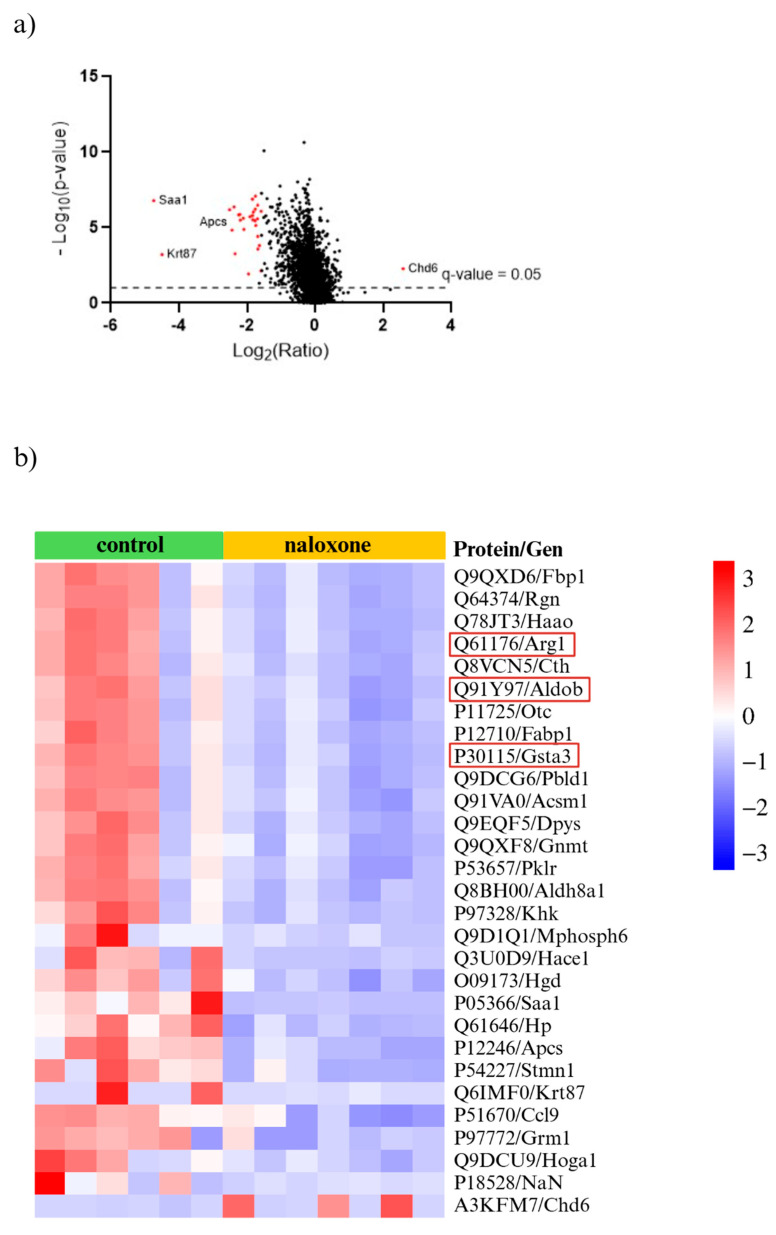
(**a**) Volcano plot for NLX-treated and non-treated mice (cut off; *p* value < 0.05, fold change ≥ 3.0 and ≤−3.0) with the most up- and downregulated proteins shown (red dots); (**b**) proteomic data obtained from aortas isolated from ApoE^−/−^ mice after NLX administration compared to non-treated. Proteins relevant to vascular dysfunction are highlighted in red (cut off; *p* value < 0.05, fold change ≥ 3.0 and ≤−3.0) (*n* = 7 in control group, *n* = 8 in NLX group).

We performed proteomic analysis of aortas collected from ApoE^−/−^ 36-week-old mice following NLX administration. Analysis identified 6869 proteins, with 587 showing significant differential expression (Figure 9a). From this subset, we selected 29 proteins with a fold change greater than 3 or less than −3 (Figure 9b). This subset included proteins involved in muscle function and structure, proteins related to cell migration and proliferation, proteins associated with the inflammatory response and immune system, proteins involved in cell death, and proteins that bind heavy metals (Supplementary Table S2).

## 4. Discussion

Current atherosclerosis treatments have remained unchanged for years, and mainly have focused on lowering cholesterol levels, omitting other important causes of atherosclerosis development [41,42,43]. Therefore, it is crucial to investigate new potential factors contributing to the development of the disease [44,45]. In this study, we undertook an investigation of the relationship between the opioid system and vascular remodelling involved in the development of atherosclerosis, with particular interest in the *Hmmr* gene role in this process. In order to specifically address the early stages of atherosclerosis development, we employed mice maintained on a chow diet, in which disease progression occurs at a slower rate. Animals were analyzed at two time points, representing an early stage (8-week-old) and a more advanced stage (36-week-old) of atherosclerosis (Supplement, Figure S3). Moreover, to explore whether opioid receptor blockade affects adaptive immunity, we analyzed splenic T-cell subpopulations in ApoE^−/−^ mice following NLX administration. Although macrophages are key mediators of atherosclerotic plaque formation, their numbers in the aortas of chow-fed ApoE^−/−^ mice are very low, making reliable visualization and quantification challenging [46,47]. In contrast, the spleen is a major secondary lymphoid organ that contains abundant naive, effector, and memory T-cells, allowing a robust assessment of systemic immune modulation.

Our previous observations and the literature indicating that the hyaluronan-mediated motility receptor participates in tissue repair, fibrosis, and cardiovascular remodelling, and that its expression can be modulated by opioid system activity, led us to investigate whether NLX administration would affect *Hmmr* expression in ApoE^−/−^ mice. We analyzed both young 8-week-old animals, representing an early stage of atherosclerosis, and 36-week-old mice, which display more advanced vascular changes. In both groups, *Hmmr* expression decreased significantly following NLX treatment. This may suggest an important role of the *Hmmr* gene in vascular remodelling at early and advanced stages of atherosclerosis development. Considering the important role of collagen in the formation of atherosclerotic plaque, we examined the mRNA expression of its two forms *Col1a1* and *Col3a1*, the content of which is highest in the vessel wall [48,49]. In the case of *Col3a1*, we observed a significant decrease in expression after NLX administration in 8-week-old mice compared to saline-treated animals of the same age, and interestingly, a significant increase in expression in 36-week-old mice. A similar trend was noted for *Col1a1*, though these changes were not statistically significant. It is known that prolonged exposure to opioid antagonists can increase tissue collagen content, as evidenced by studies with naltrexone [50,51]. Chronic elevated expression of collagen I isoforms has also been found to increase tissue stiffness and fibrosis, while increased expression of collagen III attenuates stiffness [52], and an imbalance between the synthesis of the two types affects the elasticity of veins [53]. Furthermore, age-related changes in collagen accumulation and the relative proportions of type I and III collagen have been well documented, but these changes may vary across tissue types [54]. With age, collagen type III content decreases in the heart and aorta up to the distal parts [55]; therefore, increased vascular stiffening with age is a natural consequence of ageing. We did not observe significant differences in collagen expression between 8-week-old and 36-week-old saline-treated mice indicating that the observed changes in collagen expression are not due to age differences but to NLX treatment. Changes in vascular stiffness due to affected expression and production of distinct collagen types ration upon naloxone treatment likely support plaque formation, though increased vessel rigidity has been evidenced to be a consequence of atherosclerosis development, rather than a causative factor. However, naloxone may enhance this pathological process [56]. The data suggest a relationship between the opioid system and factors involved in the development of atherosclerosis. Opioids, through their action on opioid receptors, may influence inflammatory processes and vascular remodelling, which are crucial in the development of atherosclerotic changes. Therefore, to investigate whether the blockade of opioid receptors affects the remodelling of the aortic wall, we measured the thickness of the collagen layer in the thoracic aorta of 8-week-old and 36-week-old mice following NLX treatment. We did not observe changes in the collagen layer in aorta of 8-week-old mice; however, we observed statistically significant thickening of the collagen layer in aorta of mice with advanced atherosclerosis, after NLX administration. This suggests that NLX may enhance the resistance of the aortic wall, which could have a potential protective effect in diseases such as hypertension and could contribute to inhibiting the development of aneurysms. This is supported by studies showing that treatment of aortic rings collected from pigs with collagenase led to an increase in aortic diameter and altered vessel stiffness, making it less resistant to increased blood pressure [57]. Moreover, a lower percentage of collagen in the aortic wall has been observed in aortic dissection and aortic aneurysm. The reduced collagen content in the vessel wall likely contributed to its weakening, which is a fundamental characteristic of both conditions [58]. Additionally, similar studies have been performed using another opioid receptor antagonist—naltrexone. Topical application of naltrexone to wounds in rats with type 1 diabetes resulted in enhanced collagen formation, and thus skin integrity, making the skin more difficult to tear than in vehicle-treated animals [59].

Opioids are known to modulate immune function, particularly by influencing T-cell activation, differentiation, and cytokine release, which are processes strongly implicated in the initiation and progression of atherosclerosis [12,60]. T-cells contribute significantly to vascular inflammation and can influence plaque development [12,61]. We therefore examined whether blockade of the opioid system with NLX would alter splenic T-cell subpopulations in ApoE^−/−^ mice. In addition, since *Hmmr* has been linked to tissue remodelling and immune cell activation, but its role in T-cells during atherosclerosis remains unexplored, we evaluated its expression on different T-cell subsets. This approach allowed us to investigate a novel connection between opioid signalling, immune modulation, and *Hmmr* regulation in adaptive immunity at both early and more advanced stages of the disease. We characterized subpopulations of splenic T-cells from 8-week-old and 36-week-old ApoE^−/−^ mice. Results revealed that in young mice, NLX administration did not affect a percentage of CD4^+^ T-cells and CD8^+^ T-cells. We observed significant increase in naïve, decrease in CM, but no changes in effector subpopulation. Subpopulation of effector CD4^+^ T-cells remained unchanged but we observed statistical increase in naïve and decrease in CM CD4^+^ T-cells. Moreover, we observed decrease in CM CD8^+^ T-cells but not in naive or effector CD8^+^ T-cells. Then, we indicated if NLX treatment affects HMMR expression in T-cell subpopulations. We did not observe any changes in HMMR expression on CD4^+^ T-cells and effector subpopulation. But decreased HMMR expression was observed on CD8^+^ T-cells and CD69^+^/CD4^+^ T-cells as well as in CM and naive subpopulations. Additionally, we investigated the effects of opioid receptor blockade on the T-cell population in 36-week-old mice. We did not observe changes in CD4^+^ T-cells and CD8^+^ T-cells. We also did not observe changes in naive or effector subpopulations but there was statistically significant increase in CM subpopulation. Administration of NLX did not cause changes in effector, naïve, and CM CD4^+^ T-cells. However, we observed increase in CM CD8^+^ T-cells, but not in naive or effector CD8^+^ T-cells. In line with our data, administration of cyprodime (μ-opioid receptor antagonist) to mice selected for high analgesia induced by swim stress caused increase in CM CD8^+^ T-cells population [62]. NLX administration affected HMMR expression on CD4^+^ T-cells, CD8^+^ T-cells, and CD69^+^/CD4^+^ T-cells. We also observed statistically significant increase in HMMR expression in CM, naïve, and effector subpopulations. While the results do not reveal a lot of significant changes in the T-cells population following NLX treatment, they suggest a link between opioid system blockade, HMMR expression, and the progression of atherosclerosis.

In addition to structural changes, NLX administration significantly altered the proteomic landscape of the aorta. We identified 6869 proteins, 587 of which had significantly altered expression after NLX treatment in aorta of 36-week-old ApoE^−/−^ mice compared to control. Among the proteins with most changed expressions were proteins such as fructose-bisphosphate aldolase B (Q91Y97) encoded by the *Aldob* gene, which plays a role in the proliferation of vascular smooth muscle cells. The excessive proliferation of VSMCs is a key event in the progression of vascular diseases, as it contributes to neointima formation, vascular wall thickening, and arterial stiffness, all of which are characteristic of atherosclerosis [63,64]. Another altered protein encoded by the *Gsta3* gene is glutathione S-transferase A3 (P30115). This enzyme plays a critical role in cellular detoxification processes, particularly in neutralizing reactive oxygen species (ROS) and protecting cells from oxidative stress. Since oxidative stress is a major contributor to atherogenesis, increased expression of GSTA3 may indicate an adaptive cellular response aimed at counteracting the damaging effects of lipid peroxidation and inflammation within atherosclerotic lesions [65]. Additionally, another protein with altered expression is arginase-1 (Q61176), encoded by the Arg1. This protein is a marker of M2 macrophages, which play an important role in the formation of atherosclerotic plaque and also play a key role in wound healing and tissue repair. In the context of atherosclerosis, M2 macrophages contribute to plaque stability by promoting extracellular matrix remodelling; however, they may also be involved in excessive fibrotic responses that can affect plaque composition. Arginase-1 competes with nitric oxide synthase (NOS) for L-arginine, leading to a reduction in nitric oxide (NO) production, a crucial molecule responsible for vasodilation and endothelial function. Decreased NO bioavailability can contribute to vascular dysfunction, increased oxidative stress, and heightened inflammatory responses, all of which exacerbate atherosclerotic disease progression [66,67]. However, we need to underline that despite the many beneficial effects of the opioid system in cardiovascular diseases, some studies have reported harmful effects. An example of this is research showing a connection between the opioid system and atherosclerosis in the context of depression. The comorbidity of these conditions is attributed to increased atherogenicity, insulin resistance (IR), and immune as well as oxidative stress. Neural network and logistic regression models demonstrated that severe depression in the presence of ATS/unstable angina was best predicted by interleukin-6 (IL-6), mu opioid receptor (MOR), zinc, β-endorphin, calcium, and magnesium, whereas moderate depression was associated with IL-6, zinc, MOR, β-endorphin, atherogenicity, IR, and calcium [68]. However, it should be noted that there are studies indicating that nalmefene (an antagonist of mu- and delta-opioid receptors and a partial agonist of kappa-opioid receptors) administration enhances the formation of macrophage-rich plaques in ApoE^−/−^ mice. Nalmefene also significantly increased oxLDL uptake by peritoneal macrophages in vitro, and decreased the mRNA expression of mu, delta, and kappa opioid receptors in macrophages [69].

Studies have also shown that administration of U50488H (a selective κ-opioid agonist) attenuated ischemia-induced arrhythmia in a rat model [70]. However, administration of naltrexone, an opioid antagonist, to rats with stress-induced hypercholesterolemia prevented these changes, suggesting that endogenous opioid systems play a role in the treatment of hypercholesterolemia, which is one of the causes of atherosclerosis [71].

## 5. Limitations

The research results presented above suggest that the opioid system may play a role in processes involved in the development of atherosclerosis and a potential role for the *Hmmr* gene in this disease. Further research involving usage of pharmacological agents or transgenic models will be necessary to confirm the presented findings and define potential molecular mechanism-describing changes which occur in the aorta during the development and course of atherosclerosis.

## 6. Conclusions

Our study demonstrated that blocking opioid receptors through NLX administration caused changes in the percentage of splenic T-cell subpopulations in both 8-week-old and 36-week-old mice, and also affected collagen expression and the thickness of the collagen layer in the aorta. Furthermore, NLX affected the vascular proteome in 36-week-old mice. These findings therefore suggest the involvement of the opioid system in vascular remodelling associated with the development of atherosclerosis. Moreover, the observed decrease in *Hmmr* expression following NLX administration in both young and older mice may indicate this gene’s involvement in early as well as in advanced stages of atherosclerosis.

## Data Availability

The original data presented in the study are openly available in the ProteomeXchange Consortium via the PRIDE partner repository with the dataset identifier PXD064451.

## Supplementary Materials

The following supporting information can be downloaded at: https://www.mdpi.com/article/10.3390/cells14191559/s1, Table S1: List of antibodies used for flow cytometry; Figure S1: Gating strategy of flow cytometry analysis; Figure S2: Quality control of MS runs of the aortas of control and naloxone-treated mice; Table S2: An overview of deregulated proteins, their encoding genes, and functions; Figure S3: Single control image of the aortic arch to confirm the presence of atherosclerotic plaque.
