# Supplementary material for: Opioid System Antagonism Alters Vascular Proteome and Collagen Deposition in ApoE−/− Mice
Source: Cells. 2025 Oct 8;14(19):1559. doi: 10.3390/cells14191559 (PMC12523689; doi:10.3390/cells14191559)
Supplement: Supplementary file 1 [file cells-14-01559-s001.zip › cells-3764869-supplementary.pdf]

Table S1. List of antibodies used for flow cytometry

Specificity	Fluorochrome	Clone Name	Supplier
CD3ε	PerCP	145-2C11	BioLegend
CD8a	AF700	53-6.7	BioLegend
CD4	BV750	GK 4.5	BioLegend
CD69	PEC7	H1.2F3	BioLegend
CD44	AF647	IM7	BioLegend
CD62L	PE594	MEL-14	BioLegend
CD168	CoraLite488	Polyclonal antibody	Proteintech

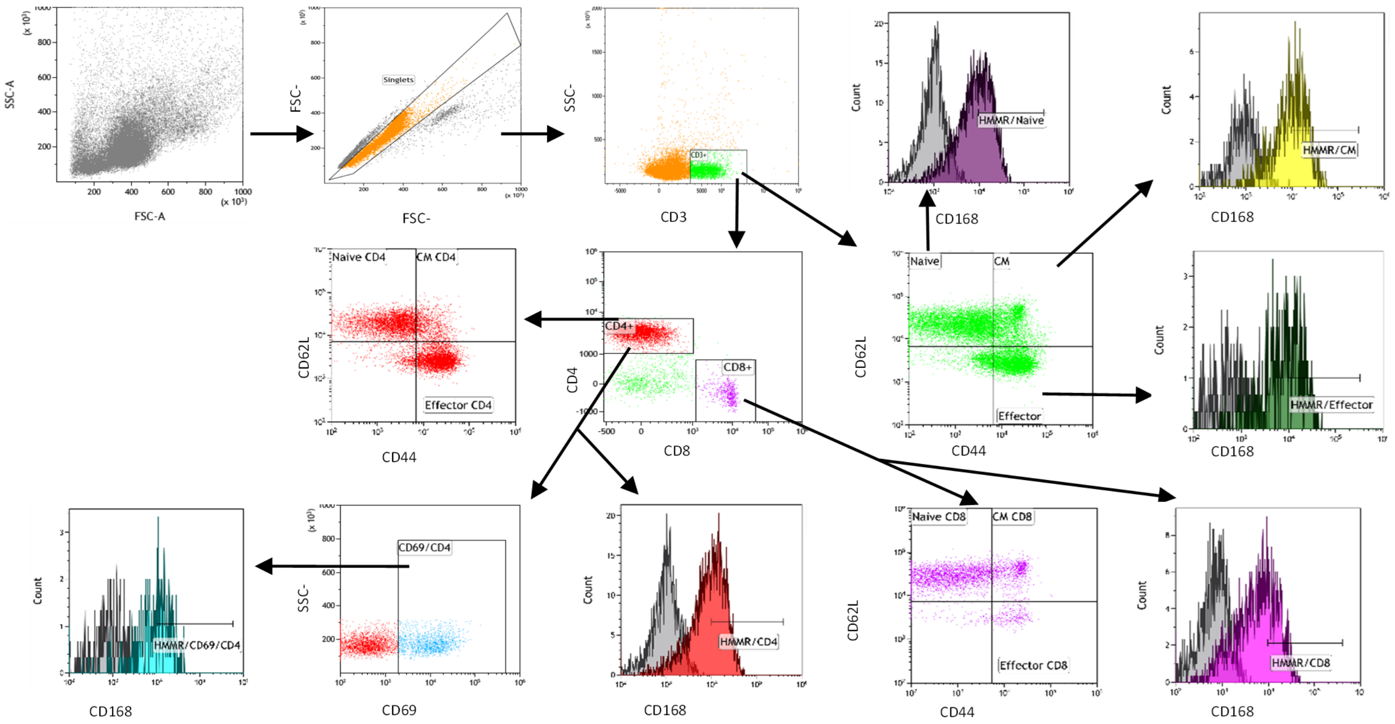

Figure S1. Gating strategy of flow cytometry analysis

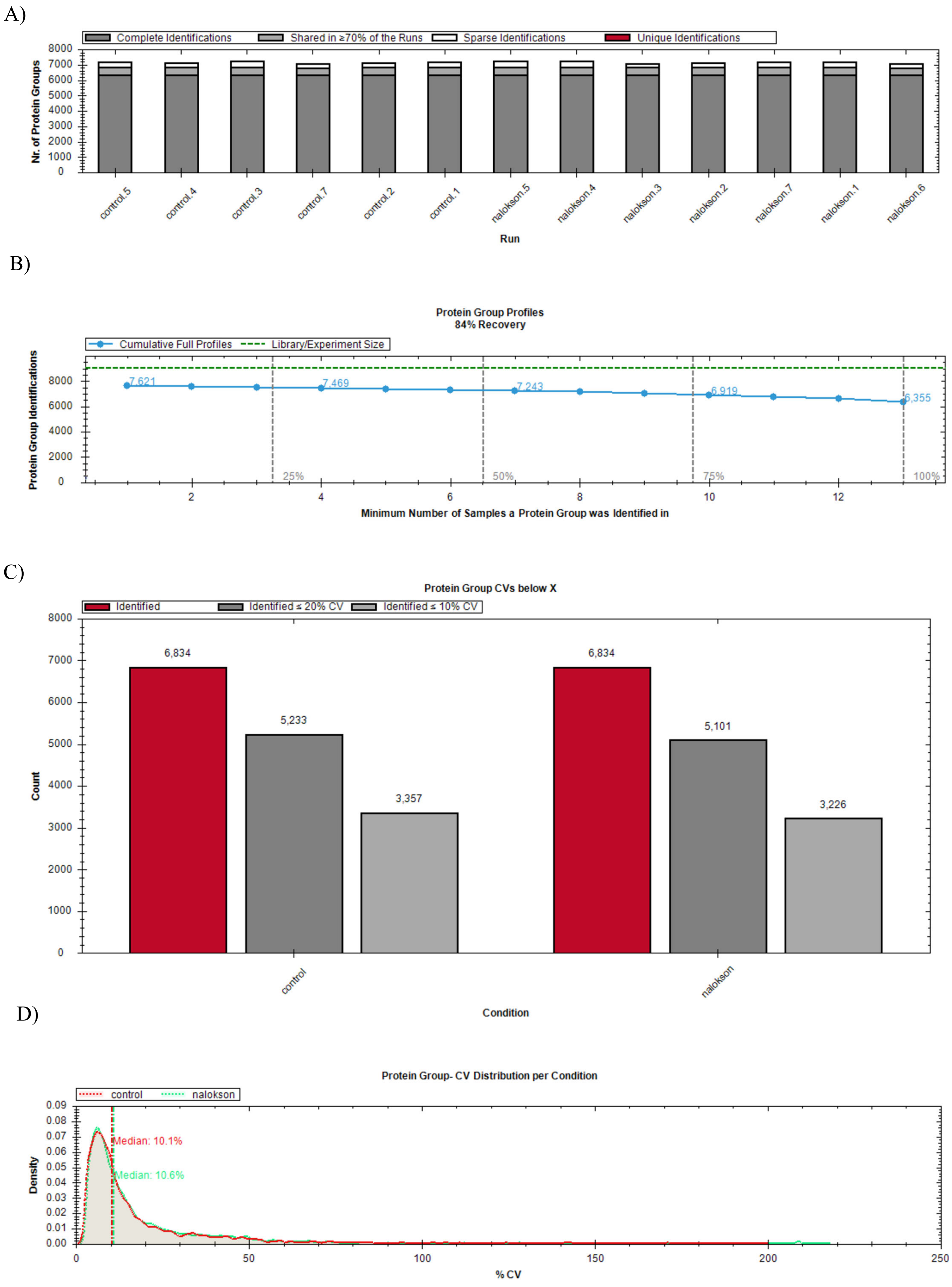

Figure S2. Quality control of MS runs of the aorta of control and naloxone-treated mice. Protein group identification details across all LC-MS runs (A). Spectral library recovery (B). Coefficient of variations (CVs) for protein groups across all biological conditions (C). Distribution of protein group CV in biological conditions (D)

Table S2. An overview of deregulated proteins, their encoding genes, and functions (functions obtained from UniProt database [1]).

Protein	Gene	Fold change	Main function	Function connected to atherosclerosis development
A3KFM7	*Chd6* (Chromodomain-helicase-DNA-binding protein 6)	6.1	DNA-dependent ATPase that plays a role in chromatin remodeling. Regulates transcription by disrupting nucleosomes in a largely non-sliding manner which strongly increases the accessibility of chromatin.	-
P30115	*Gsta3* (Glutathione S-transferase A3)	-3.01	Conjugation of reduced glutathione to a wide number of exogenous and endogenous hydrophobic electrophiles. Catalyzes isomerization reactions that contribute to the biosynthesis of steroid hormones.	The expression of *Gsta3* mRNA in the aortic arch of ApoE$^{-/-}$ mice increased in the period preceding the development of atherosclerotic lesions (age from 6 to 12 weeks), whereas it decreased inversely proportional to the development of lesions (between 12 and 34 weeks) [14].
P18528	Ig heavy chain V region 6.96	-3.01		-
P97772	*Grm1* (Metabotropic glutamate receptor 1)	-3.11	G-protein coupled receptor for glutamate. Ligand binding causes a conformation change that triggers signaling via guanine nucleotide-binding proteins (G proteins) and modulates the activity of down-stream effectors. Signaling activates a phosphatidylinositol-calcium second messenger system.	-
Q9DCU9	*Hoga1* (4-hydroxy-2-oxoglutarate aldolase, mitochondrial)	-3.21	Catalyzes the final step in the metabolic pathway of hydroxyproline.	-
P51670	*Ccl9* (C-C motif chemokine 9)	-3.22	Monokine with inflammatory, pyrogenic and chemokinetic properties. Circulates at high concentrations in the blood of healthy animals. Binding to a high-affinity receptor activates calcium release in neutrophils.	-
O09173	*Hgd* (Homogentisate 1,2-dioxygenase)	-3.22	Catalyzes the conversion of homogentisate to maleylacetoacetate.	-
Q8BH00	*Aldh8a1* (2-aminomuconic semialdehyde dehydrogenase)	-3.23	Catalyzes the NAD-dependent oxidation of 2-aminomuconic semialdehyde of the kynurenine metabolic pathway in L-tryptophan degradation.	-

Q9DCG6	*Pbld1* (Phenazine biosynthesis-like domain-containing protein 1)	-3.34	Involved in maintenance of gastrointestinal epithelium.	-
P53657	*Pklr* (Pyruvate kinase PKLR)	-3.37	Pyruvate kinase that catalyzes the conversion of phosphoenolpyruvate to pyruvate with the synthesis of ATP, and which plays a key role in glycolysis.	-
Q91VA0	*Acsm1* (Acyl-coenzyme A synthetase ACSM1, mitochondrial)	-3.38	Catalyzes the activation of fatty acids by CoA to produce an acyl-CoA, the first step in fatty acid metabolism.	-
Q9QXF8	*Gnmt* (Glycine N-methyltransferase)	-3.40	Catalyzes the methylation of glycine by using S-adenosylmethionine (AdoMet) to form N-methylglycine (sarcosine) with the concomitant production of S-adenosylhomocysteine (AdoHcy).	$ApoE^{-/-}/Gnmt^{-/-}$ mice had significantly increased development of atherosclerotic lesions in the aortic roots compared with $ApoE^{-/-}$ mice. Furthermore, $ApoE^{-/-}$ $GNMT^{-/-}$ mice showed increased levels of *IL-6*, *TNF-α, MCP-1,* and *MIP-2* in serum and aorta and higher expression of *VCAM-1* and *iNOS* in aortas than $ApoE^{-/-}$ mice [20].
Q91Y97	*Aldob* (Fructose-bisphosphate aldolase B)	-3.51	Catalyzes the aldol cleavage of fructose 1,6-biphosphate to form two triosephosphates dihydroxyacetone phosphate and D-glyceraldehyde 3-phosphate in glycolysis as well as the reverse stereospecific aldol addition reaction in gluconeogenesis.	The knockdown of *AldoB* expression prevented fructose-induced methylglyoxal overproduction and vascular smooth muscle cells proliferation [19].
Q61176	*Arg1* (Arginase-1)	-3.56	Key element of the urea cycle converting L-arginine to urea and L-ornithine. Plays a role in the immune response of alternatively activated or M2 macrophages in processes such as wound healing and tissue regeneration	*Agr1* gene expression was significantly higher in peritoneal macrophages from 28-week-old Fcγ receptor-deficient mice ($\gamma^{-/-}ApoE^{-/-}$) than in $ApoE^{-/-}$ mice [17].
P11725	*Otc* (Ornithine transcarbamylase, mitochondrial)	-3.58	Catalyzes the second step of the urea cycle, the condensation of carbamoyl phosphate with L-ornithine to form L-citrulline.	Data from databases obtained during two studies conducted in France (MONICA and EVA) indicated that in men, the frequency of the OTC single nucleotide polymorphism rs5963409 was higher in hypertensive than in normotensive subjects [16].
Q61646	*Hp* (Haptoglobin)	-3.58	Haptoglobin captures, and combines with free plasma hemoglobin to allow hepatic recycling of heme iron and to prevent kidney damage. Haptoglobin also acts as an antioxidant, has antibacterial activity and plays a role in modulating many aspects of the acute phase response	Creation a murine type 2 Hp allele and targeted its insertion to the Hp locus by homologous recombination coused increased iron, lipid peroxidation and macrophage accumulation in plaques of $ApoE^{-/-}$ Hp 2-2 mice what suggest that the Hp genotype plays a critical

				role in the oxidative and inflammatory response to intraplaque hemorrhage [6].
Q8VCN5	*Cth* (Cystathionine gamma-lyase)	-3.77	Catalyzes the last step in the trans-sulfuration pathway from L-methionine to L-cysteine in a pyridoxal-5'-phosphate (PLP)-dependent manner.	Studies have shown that in the aortas of *cth*$^{SMC-/-}$ mice (VSMC-specific *cth* knockout mice), En-face Oil Red O staining showed that the number of aortic plaques increased by about 76% in comparison to the control [18].
Q9D1Q1	*Mphosph6* (M-phase phosphoprotein 6)	-3.89	RNA-binding protein that associates with the RNA exosome complex. Plays a role in recruiting the RNA exosome complex to pre-rRNA	Genetic polymorphisms of MPHOSPH6 (rs1056654) were associated with a decreased risk of coronary artery disease in Chinese Han population [8].
Q9EQF5	*Dpys* (Dihydropyrimidinase)	-4.27	Catalyzes the second step of the reductive pyrimidine degradation, the reversible hydrolytic ring opening of dihydropyrimidines.	-
P97328	*Khk* (Ketohexokinase)	-4.31	Catalyzes the phosphorylation of the ketose sugar fructose to fructose-1-phosphate.	Mice deficient in ketohexokinase (KHK) showed reduce features of non-alcoholic steatohepatitis (NASH) on a high-fat/high-fructose diet. KHK inhibition reduced lipogenic gene expression in the presence of high fructose/glucose. Moreover, activated myofibroblasts exhibit reduced expression of fibrogenic genes when treated with a KHK inhibitor. The KHK inhibitor also decreases hepatic accumulation of lipogenic fructose derivatives and reduces glycolysis in human liver tissue [25].
P12710	*Fabp1* (Fatty acid-binding protein)	-4.59	Plays a role in lipoprotein-mediated cholesterol uptake in hepatocytes. Binds cholesterol. Binds free fatty acids and their coenzyme A derivatives, bilirubin, and some other small molecules in the cytoplasm.	The study cohort included 479 Chinese subjects who underwent carotid intima-media thickness (IMT) measurement. Serum A-FABP levels were positively associated with carotid IMT in both men and women [27].
Q9QXD6	*Fbp1* (Fructose-1,6-bisphosphatase 1)	-4.62	Catalyzes the hydrolysis of fructose 1,6-bisphosphate to fructose 6-phosphate in the presence of divalent cations, acting as a rate-limiting enzyme in gluconeogenesis. Plays a role in regulating glucose sensing and insulin secretion of pancreatic beta-cells.	Studies have shown Selective knockdown of FBP1 significantly enhanced the migration, and proliferation of hESC-ECs (human embryonic stem cell-derived endothelial cells), implying activation of angiogenesis. Moreover, FBP1 inhibition partially reversed the inhibitory effect of retinoic acid on angiogenesis [24].
Q78JT3	*Haao* (3-hydroxyanthranilate 3,4-dioxygenase)	-4.74	Catalyzes the oxidative ring opening of 3-hydroxyanthranilate to 2-amino-3-carboxymuconate semialdehyde, which spontaneously cyclizes to quinolinate.	Research demonstrated that treatment of Ldlr-/- mice with the HAAO inhibitor (NCR-631) significantly reduced atherosclerotic lesion area in the aortic arch compared to PBS controls. NCR-631 treatment also

				resulted in lower plasma levels of total cholesterol and triglycerides compared to controls [23].
Q3U0D9	*Hace1*<br>(E3 ubiquitin-protein ligase HACE1)	-5.10	Acts as a regulator of Golgi membrane dynamics during the cell cycle: recruited to Golgi membrane by Rab proteins and regulates postmitotic Golgi membrane fusion.	-
Q64374	*Rgn*<br>(Regucalcin)	-5.20	Gluconolactonase with low activity towards other sugar lactones, including gulonolactone and galactonolactone. Catalyzes a key step in ascorbic acid (vitamin C) biosynthesis.	Liver regucalcin gene expression is stimulated through action of insulin in liver cells and decreased in type I diabetic model animals. Overexpreesion of regucalcin reveals hepatic insulin resistance, decreased liver triglyceride, total cholesterol and glycogen contents in the liver of rats, inducing a hyperlipidemia. Liver leptin and adiponectin mRNA expressions are decreased by overexpression of regucalcin. Deficiency of regucalcin induces an impairment of glucose tolerance and liver lipid accumulation in mice, and it is associated with the development and progression of nonalcoholic fatty liver disease and fibrosis in human patients [26].
P54227	*Stmn1*<br>(Stathmin)	-5.42	Involved in the regulation of the microtubule (MT) filament system by destabilizing microtubules. Prevents assembly and promotes disassembly of microtubules.	-
P12246	*Apcs*<br>(Serum amyloid P-component)	-5.70	Belonging to the pentraxin family of proteins, which has a characteristic pentameric organization.	-
Q6IMF0	*Krt87*<br>(Keratin, type II cuticular 87)	-22.55	Predicted to be a structural constituent of skin epidermis.	-
P05366	*Saa1*<br>(serum amyloid A1)	-26.78	Major acute phase reactant	ApoE$^{-/-}$ mice overexpressed murine SAA1 exhibited modest but persistent increase in SAA that contributed to increased atherosclerosis via increased inflammatory cell infiltration [29].<br>Rag1$^{-/-}$ApoE$^{-/-}$ and ApoE$^{-/-}$ mice injected with adenoviral vector encoding human SAA1 had increased atherosclerosis compared with controls [30].

A) B)

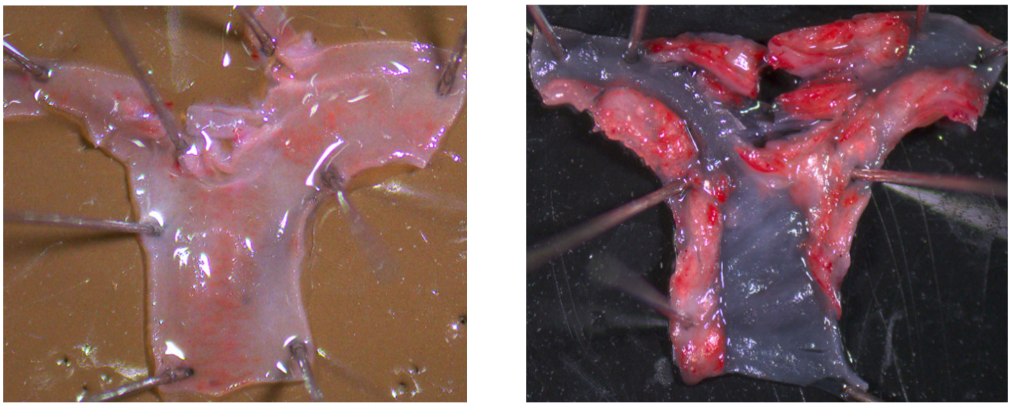

Figure S3. Single control image of the aortic arch to confirm the presence of atherosclerotic plaque. A) 8-week-old NaCl; B) 36- week-old NaCl.

[1] UniProt: the Universal Protein Knowledgebase in 2023. Nucleic Acids Res 2023;51:D523–31. https://doi.org/10.1093/nar/gkac1052.

[2] Oner T, Arslan C, Yenmis G, Arapi B, Tel C, Aydemir B, et al. Association of NFKB1A and microRNAs variations and the susceptibility to atherosclerosis. J Genet 2017;96:251–9. https://doi.org/10.1007/S12041-017-0768-9.

[3] Carlson CS, Heagerty PJ, Nord AS, Pritchard DK, Ranchalis J, Boguch JM, et al. TagSNP evaluation for the association of 42 inflammation loci and vascular disease: evidence of IL6, FGB, ALOX5, NFKBIA, and IL4R loci effects. Hum Genet 2007;121:65–75. https://doi.org/10.1007/S00439-006-0289-8.

[4] Rezq AA, Mahmoud MY. Preventive effect of wheat germ on hypercholesteremic and atherosclerosis in rats fed cholesterol-containing diet. Pakistan Journal of Nutrition 2011;10:424–32. https://doi.org/10.3923/pjn.2011.424.432.

[5] Stachowicz A, Olszanecki R, Suski M, Wiśniewska A, Kuś K, Białas M, et al. Quantitative proteomics reveals decreased expression of major urinary proteins in the liver of apoE/eNOS-DKO mice. Clin Exp Pharmacol Physiol 2018;45:711–9. https://doi.org/10.1111/1440-1681.12927.

[6] Levy AP, Levy JE, Kalet-Litman S, Miller-Lotan R, Levy NS, Asaf R, et al. Haptoglobin Genotype Is a Determinant of Iron, Lipid Peroxidation, and Macrophage Accumulation in the Atherosclerotic Plaque 2006. https://doi.org/10.1161/01.ATV.0000251020.24399.a2.

[7] Chen Y, Yang M, Zhang M, Wang H, Zheng Y, Sun R, et al. Single-Cell Transcriptome Reveals Potential Mechanisms for Coronary Artery Lesions in Kawasaki Disease. Arterioscler Thromb Vasc Biol 2024;44:866–82. https://doi.org/10.1161/ATVBAHA.123.320188.

[8] Song Y, Yan M, Li J, Li J, Jin T, Chen C. Association between TNIP1, MPHOSPH6 and ZNF208 genetic polymorphisms and the coronary artery disease risk in Chinese Han population n.d.

[9] Liu Y, Zhao Y, Hayek T, Chen YE, Rom O, Shukha Y, et al. Dysregulated oxalate metabolism is a driver and therapeutic target in atherosclerosis. CellReports 2021;36:109420. https://doi.org/10.1016/j.celrep.2021.109420.

[10] Boehme B, Schelski N, Makridakis M, Henze L, Vlahou A, Lang F, et al. Role of Cytosolic Serine Hydroxymethyl Transferase 1 (SHMT1) in Phosphate-Induced Vascular Smooth Muscle Cell Calcification. Kidney Blood Press Res 2018;43:1212–21. https://doi.org/10.1159/000492248.

[11] Gu X, Yu Z, Qian T, Jin Y, Xu G, Li J, et al. Transcriptomic analysis identifies the shared diagnostic biomarkers and immune relationship between Atherosclerosis and abdominal aortic aneurysm based on fatty acid metabolism gene set. Front Mol Biosci 2024;11. https://doi.org/10.3389/fmolb.2024.1365447.

[12] Kisucka J, Chauhan AK, Patten IS, Yesilaltay A, Neumann C, Van Etten RA, et al. Peroxiredoxin1 prevents excessive endothelial activation and early atherosclerosis. Circ Res 2008;103:598–605. https://doi.org/10.1161/CIRCRESAHA.108.174870.

[13] Madrigal-Matute J, Fernandez-Garcia CE, Blanco-Colio LM, Burillo E, Fortuño A, Martinez-Pinna R, et al. Thioredoxin-1/peroxiredoxin-1 as sensors of oxidative stress mediated by NADPH oxidase activity in atherosclerosis. Free Radic Biol Med 2015;86:352–61. https://doi.org/10.1016/J.FREERADBIOMED.2015.06.001.

[14] 't Hoen PAC, Van Der Lans CAC, Eck M Van, Bijsterbosch MK, Van Berkel TJC, Twisk J. Aorta of ApoE-Deficient Mice Responds to Atherogenic Stimuli by a Prelesional Increase and Subsequent Decrease in the Expression of Antioxidant Enzymes 2003. https://doi.org/10.1161/01.RES.0000082978.92494.B1.

[15] AGXT2 Polymorphism and CHD Risk n.d.

[16] Dumont J, Meroufel D, Bauters C, Hansmannel F, Bensemain F, Cottel D, et al. Association of Ornithine Transcarbamylase Gene Polymorphisms With Hypertension and Coronary Artery Vasomotion. Am J Hypertens 2009;22:993–1000. https://doi.org/10.1038/AJH.2009.110.

[17] Mallavia B, Oguiza A, Lopez-Franco O, Recio C, Ortiz-Muñ Oz G. Gene Deficiency in Activating Fcc Receptors Influences the Macrophage Phenotypic Balance and Reduces Atherosclerosis in Mice. PLoS One 2013;8:66754. https://doi.org/10.1371/journal.pone.0066754.

[18] Chen Z, Ouyang C, Zhang H, Gu Y, Deng Y, Du C, et al. Vascular smooth muscle cell-derived hydrogen sulfide promotes atherosclerotic plaque stability via TFEB (transcription factor EB)-mediated autophagy 2022. https://doi.org/10.1080/15548627.2022.2026097.

[19] Cao W, Chang T, Li X qiang, Wang R, Wu L. Dual effects of fructose on ChREBP and FoxO1/3α are responsible for AldoB up-regulation and vascular remodelling. Clin Sci 2017;131:309–25. https://doi.org/10.1042/CS20160251.

[20] Chen CY, Ching LC, Liao YJ, Yu Y Bin, Tsou CY, Shyue SK, et al. Deficiency of Glycine N-methyltransferase aggravates atherosclerosis in apolipoprotein E-null mice. Molecular Medicine 2012;18:744–52. https://doi.org/10.2119/molmed.2011.00396.

[21] Van Dongen K, Leleu D, Pilot T, Jalil A, Mangin L, Ménégaut L, et al. Atheroma plaque microenvironment stimulates kynurenine production by macrophages to induce endothelial adhesion molecules in the context of atherogenesis 2023. https://doi.org/10.1101/2023.07.19.549799.

[22] Dong J, Song C, Zhang L, Feng X, Feng R, Lu Q, et al. Identified key genes related to carotid atheroma plaque from gene expression chip. Artif Cells Nanomed Biotechnol 2017;45:1132–7. https://doi.org/10.1080/21691401.2016.1216858.

[23] Berg M, Polyzos KA, Agardh H, Baumgartner R, Forteza MJ, Kareinen I, et al. 3-Hydroxyanthralinic acid metabolism controls the hepatic SREBP/lipoprotein axis, inhibits inflammasome activation in macrophages, and decreases atherosclerosis in Ldlr-/- mice. Cardiovasc Res 2020;116:1948–57. https://doi.org/10.1093/CVR/CVZ258.

[24] Yang Z, Yu M, Li X, Tu Y, Wang C, Lei W, et al. Retinoic acid inhibits the angiogenesis of human embryonic stem cell-derived endothelial cells by activating FBP1-mediated gluconeogenesis. Stem Cell Res Ther 2022;13. https://doi.org/10.1186/S13287-022-02908-X.

[25] Shepherd EL, Saborano R, Northall E, Matsuda K, Ogino H, Yashiro H, et al. Ketohexokinase inhibition improves NASH by reducing fructose-induced steatosis and fibrogenesis. JHEP Rep 2020;3. https://doi.org/10.1016/J.JHEPR.2020.100217.

[26] Yamaguchi M, Murata T. Involvement of regucalcin in lipid metabolism and diabetes. Metabolism 2013;62:1045–51. https://doi.org/10.1016/J.METABOL.2013.01.023.

[27] Yeung D, Xu A, Cheung C, Wat N, Yau M, Fong C, et al. Serum Adipocyte Fatty Acid-Binding Protein Levels Were Independently Associated With Carotid Atherosclerosis 2007. https://doi.org/10.1161/ATVBAHA.107.146274.

[28] Webb NR, De Beer MC, Wroblewski JM, Ji A, Bailey W, Shridas P, et al. Deficiency of Endogenous Acute-Phase Serum Amyloid A Protects apoE-/- Mice From Angiotensin II-Induced Abdominal Aortic Aneurysm Formation. Arterioscler Thromb Vasc Biol 2015;35:1156–65. https://doi.org/10.1161/ATVBAHA.114.304776.

[29] Dong Z, Wu T, Qin W, An C, Wang Z, Zhang M, et al. Serum amyloid A directly accelerates the progression of atherosclerosis in apolipoprotein E-deficient mice. Mol Med 2011;17:1357–64. https://doi.org/10.2119/MOLMED.2011.00186.

[30] Thompson JC, Jayne C, Thompson J, Wilson PG, Yoder MH, Webb N, et al. A brief elevation of serum amyloid A is sufficient to increase atherosclerosis. J Lipid Res 2015;56:286–93. https://doi.org/10.1194/JLR.M054015.
